# Effects of *Aspergillus fumigatus* Conidia on Apoptosis and Proliferation in an In Vitro Model of the Lung Microenvironment

**DOI:** 10.3390/microorganisms9071435

**Published:** 2021-07-02

**Authors:** Hisako Kushima, Toshiyuki Tsunoda, Taichi Matsumoto, Yoshiaki Kinoshita, Koichi Izumikawa, Senji Shirasawa, Masaki Fujita, Hiroshi Ishii

**Affiliations:** 1 Department of Respiratory Medicine, Faculty of Medicine, Fukuoka University, Fukuoka 814-0180, Japan; mfujita@fukuoka-u.ac.jp; 2 Department of Respiratory Medicine, Fukuoka University Chikushi Hospital, Fukuoka 818-8502, Japan; y3kinoshita@fukuoka-u.ac.jp (Y.K.); hishii@fukuoka-u.ac.jp (H.I.); 3 Department of Cell Biology, Faculty of Medicine, Fukuoka University, Fukuoka 814-0180, Japan; tsunoda@fukuoka-u.ac.jp (T.T.); sshirasa@fukuoka-u.ac.jp (S.S.); 4 Department of Central Research Institute for Advanced Molecular Medicine, Fukuoka University, Fukuoka 814-0180, Japan; 5 Department of Pharmaceutical Sciences, Faculty of Drug Informatics and Translational Research, Fukuoka University, Fukuoka 814-0180, Japan; tmatsumoto@fukuoka-u.ac.jp; 6 Department of Infectious Diseases, Nagasaki University Graduate School of Biomedical Sciences, Nagasaki 852-8501, Japan; koizumik@nagasaki-u.ac.jp

**Keywords:** *Aspergillus fumigatus*, lung epithelial cells, fibroblast, apoptosis, proliferation

## Abstract

Background/Aim: *Aspergillus* is often detected in respiratory samples from patients with chronic respiratory diseases, including pulmonary fibrosis, suggesting that it can easily colonize the airways. To determine the role of *Aspergillus* colonization in pulmonary fibrosis, we cultured human lung epithelial A549 cells or murine embryo fibroblast NIH/3T3 cells with *Aspergillus* conidia in 3D floating culture representing the microenvironment. Materials and Methods: Cells were cultured in two-dimensional (2D) and three-dimensional floating (3DF) culture with heat-inactivated *Aspergillus fumigatus* (AF) 293 conidia at an effector-to-target cell ratio of 1:10 (early-phase model) and 1:100 (colonization model), and RNA-sequencing and Western blots (WB) were performed. Results: AF293 conidia reduced A549 cell growth in 2D and 3DF cultures and induced apoptosis in A549 spheroids in 3DF culture. RNA-sequencing revealed the increased expression of genes associated with interferon-mediated antiviral responses including MX dymamin-like GTPase 1 (MX1). Interestingly, the decreased expression of genes associated with the cell cycle was observed with a high concentration of AF293 conidia. WB revealed that epithelial-mesenchymal transition was not involved. Notably, AF293 conidia increased NIH/3T3 growth only in 3DF culture without inducing an apoptotic reaction. RNA-sequencing revealed the increased expression of genes associated with interferon signalling, including MX2; however, the decreased expression of genes associated with the cell cycle was not observed. Conclusions: AF affects both apoptosis of epithelial cells and the growth of fibroblasts. A deeper understanding of the detailed mechanisms underlying *Aspergillus*-mediated signaling pathway in epithelial cells and fibroblasts will help us to understand the lung microenvironment.

## 1. Introduction

*Aspergillus* species (spp.) are universally and widely present in the environment, including air-conditioning units, compost, and damp or flood-damaged housing and hospital building projects [1]. Although invasive pulmonary aspergillosis is a fatal disease caused by *Aspergillus* spp. in immunocompromised hosts, airway colonization by *Aspergillus* spp. is observed in approximately half of patients in an adult pneumology ward despite no typical symptoms of aspergillosis [2,3]. Among cases of chronic respiratory disease, such as intestinal pneumonia/pulmonary fibrosis, 71% of patients did not develop any kind of aspergillosis despite *Aspergillus* colonization in the airway [2,3], suggesting an unknown association between *Aspergillus* colonization and the development and progression of lung fibrosis other than aspergillosis.

Idiopathic pulmonary fibrosis is well known to be a refractory and progressive lung disease with a poor prognosis [4,5]. Several molecular mechanisms have been hypothesized to underlie the development of pulmonary fibrosis, such as apoptosis and/or epithelial-mesenchymal transition (EMT) of pulmonary epithelial cells; however, most upstream triggers of the disease remain unclear [6,7,8,9,10,11].

Among *Aspergillus* spp., *Aspergillus fumigatus* (AF) is reported to be more evident in pulmonary fibrosis [12]. A previous in vitro study addressed the association between AF and pulmonary fibrosis using AF conidia and lung epithelial cells; however, the AF conidia used these studies were heat inactivated at high temperature (90,120 °C) or killed [13,14]. Furthermore, these studies were only performed in a two-dimensional (2D) culture. In the present study, to examine the interaction between AF conidia and epithelial cells or fibroblasts in a pathophysiological state, we treated AF conidia at low temperature (70 °C) and applied both 2D and three-dimensional (3D) floating (3DF) co-culture methods, which seem to resemble the lung microenvironment [15], at an effector-to-target cell ratio of 1:10 (early-phase model) and 1:100 (colonization model).

## 2. Materials and Methods

### 2.1. Antibodies

An EMT antibody sampler kit, including anti-ZO-1 antibody, anti-vimentin antibody and anti-e-cadherin, was purchased from Cell Signaling (Tokyo, Japan). Anti-actin antibody was purchased from Sigma-Aldrich (St. Louis, MO, USA).

### 2.2. Suspension of AF293 Conidia

*A. fumigatus* (AF293), a widely used reference strain, was kindly provided by the Department of Infectious Diseases, Nagasaki University Graduate School of Biomedical Sciences, Nagasaki, Japan. The strain was isolated from the lung of a leukopenic patient who died with invasive pulmonary aspergillosis at the university. For experiments, cultures of AF293 isolate were maintained in potato dextrose agar. After five days at room temperature, the cells were harvested. The harvested cells were suspended in 5 mL of phosphate-buffered saline (PBS) with 1% Tween 20 and then filtered through a cell strainer. In this process, the agar and hyphae were removed, and only the conidia were collected. The conidia were precipitated by centrifugation at 3500 rpm for 5 min, and then the supernatant was discarded. After washing twice in 5 mL of PBS with 1% Tween 20, the sample was suspended in an appropriate amount of PBS (about 4 mL) and adjusted to 10^8^ cells per milliliter of PBS. The conidia were then inactivated by heating at 70 °C for 1 h. Killing was determined by plating on agar.

### 2.3. A Two-Dimensional (2D) Proliferation Assay

A549 cells (human alveolar adenocarcinoma cell line) as a model of type II pneumocytes and mouse NIH/3T3 fibroblasts (200,000 cells) were seeded in 10 cm dishes (Corning Inc., Corning, NY, USA) [16]. Cells were cultured at 37 °C in 5% CO_2_ with or without heat-inactivated AF293 conidia at an effector-to-target cell (E:T) ratio of 1:10 and 1:100, as described previously [17]. Images of cells were taken using a BZ-X700 (Keyence, Osaka, Japan) on days 0 and 3. The cells were also trypsin-treated and trypan blue-stained on day 3, and the number of cells was counted.

### 2.4. Three-Dimensional Floating (3DF) Cell Culture

A549 and mouse NIH/3T3 cells (2000 cells/well) were seeded in round-bottomed 96-well plates with ultra-low-attachment surfaces (Corning Inc.). Cells were cultured in a CO_2_ incubator with or without heat-inactivated AF293 conidia at an E:T ratio of 1:10 and 1:100, as described previously [15,17,18,19]. Images of cells were taken using a BZ-X700 (Keyence), and the spheroid area was measured using a BZ Analyzer (Keyence), as described previously [15,20]. The growth rates of tumor spheroids were calculated based on the changes in the spheroid area on days 3 and 7.

### 2.5. Detection of Apoptotic Cells in Spheroids

Apoptotic cells were stained on day 7 using a CellEvent^TM^ Caspase-3/7 Green Detection Kit (Invitrogen, Thermo Fisher Scientific, Waltham, MA, USA) according to the manufacturer’s instructions. Cells were imaged using a BZ-X700 (Keyence). Integration of the fluorescence intensity/area were measured using a BZ-X700 Analyzer (Keyence).

### 2.6. Western Blotting

Cells were lysed in Radioimmnunoprecipitation assay buffer (RIPA) buffer (50 mM Tris–HCl, pH 7.5, 150 mM NaCl, 1% NP40, 0.5% sodium deoxycholate, 0.1% sodium dodecyl sulfate, and protease inhibitor cocktail [Roche, Basel, Switzerland]) and subjected to immunoblotting, as described previously [20]. Quantitative analyses of the immunoblots were performed using the ImageJ 1.52a software program (National Institutes of Health, Bethesda, MD, USA).

### 2.7. RNA Sequencing Analysis (RNA-Seq)

RNA-seq was performed by Kyushu Prosearch LLP (Fukuoka, Japan). In brief, total RNA was extracted from A549 cells and NIH/3T3 cells at day 3 using TRIzol reagent (Invitrogen, Thermo Fisher Scientific) according to the manufacturer’s instruction. The RNA quality was examined using an RNA 6000 Pico kit (Agilent, Santa Clara, CA, USA). An amount of 500 ng total RNA was used for RNA-seq library preparation with NEBNext rRNA Depletion Kit and NEBNext Ultra Directional RNA Library Prep Kit (New England Biolabs, Ipswich, MA, USA). Paired-end sequencing (2 × 36 bases) was performed with a NextSeq500 (Illumina, San Diego, CA, USA). Reads were mapped on hg19 human reference genome and quantified using CLC Genomics Workbench v10.1.1 (Qiagen, Redwood City, CA, USA).

### 2.8. Real-Time Quantitative Reverse Transcription-Polymerase Chain Reaction (qRT-PCR)

Total RNA was extracted using TRIzol (Thermo Fisher Scientific K.K., Tokyo, Japan) before synthesis of cDNA using SuperScript IV VILO Master Mix (Thermo Fisher Scientific K.K.). cDNA was amplified using THUNDERBIRD^®^ SYBR qPCR Mix (TOYOBO CO., LTD., Osaka, Japan) and gene-specific primers, as described previously [20]. The gene amplification was monitored by quantitative polymerase chain reaction (PCR) was performed using Step One Plus real-time PCR System (Thermo Fisher Scientific K.K.). The PCR primers used for each of the genes were as follow: MX1-Sense, 5′-GGCTGTTTACCAGACTCCGACA-3′ and MX1-Antisense, 5′-CAC AAAGCCTGGCAGCTCTCTA-3′; OASL-Sense, 5′-CACCATTGTGCCTGCCTACA-3′ and OASL-Antisense, 5′-TCACGAAATTTCTCTGCAGC-3′; TRIM22-Sense, 5′-GGATCGTCAGTAGAGATGCTGC-3′ and TRIM22-Antisense, 5′-GAACTTGCAGCATCCCACTCAG-3′; RSAD2-Sense, 5′-AGG AAGGTGAGGTGAATGTG-3′ and RSAD2-Antisense, 5′-TGGCTTCTTCAATGTCCAGC-3′; GAPDH-Sense, 5′-TCTGCTCCTCCTGTTCGAACA-3′ and GAPDH-Antisense, 5′-AAAAGCAGCCCTGGTGACC-3′.

### 2.9. Ontology Analyses

Ontology analyses were performed using ConsensusPathDB-human according to interactions [21]. In brief, a gene set analysis was performed by an over-representation analysis. The gene identifier was set to the gene symbol, and pathways were defined by the Reactome Pathway Database.

### 2.10. Statistical Analyses in Cell Culture Experiments

Statistical analyses were performed using unpaired two-tailed Student’s *t*-tests. All *p*-values less than 0.05 were considered statistically significant.

## 3. Results

### 3.1. AF293 Conidia Suppress the Growth of A549 Cells

**Figure 1:**
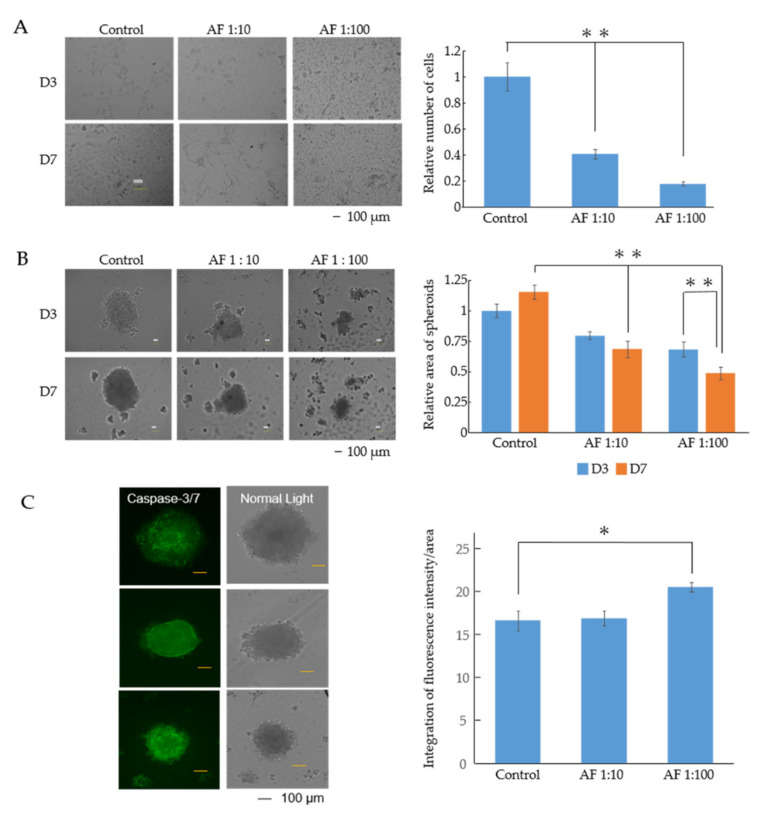
Heat-inactivated AF293 conidia suppress the growth of A549 cells. (**A**) Morphological changes and numbers of A549 cells with AF293 conidia at ratios of 1:10 and 1:100 on days 3 and 7 in 2D culture. AF 1:10 and AF 1:100 represents the number of AF293 conidia are 10- and 100-fold higher than that of A549 cells. D3: day 3, D7: day 7, Scale bar: 100 μm. Right panel represents the relative number of cells. The mean ± standard deviation (SD) from three technical replicates is shown. ** *p* < 0.001. (**B**) Morphological changes and relative area of A549 spheroids with AF293 conidia at ratios of 1:10 and 1:100 on days 3 and 7 in 3D culture. The right panel represents the area of spheroids. The mean ± SD from three technical replicates is shown. ** *p* < 0.001. (**C**) Representative signals for caspase-3/7 in A549 spheroids from three technical replicates (scale bar: 100 µm). The right panel represents the integration of fluorescence intensity/area. The mean ± SD from three technical replicates is shown. * *p* < 0.01. All data are representative of three independent experiments.

The control A549 cells became monolayered and adherent as the days passed. However, no such morphological changes occurred in the A549 with AF293 heat-inactivated conidia (Figure 1A). The numbers of AF293 conidia used for this experiment were 10-fold (1:10) and 100-fold (1:100) higher than those of A549 cells. In 2D culture, the relative numbers of cells with AF293 conidia at ratios of 1:10 and 1:100 were 0.407- and 0.176-fold smaller than those of the control, respectively (Figure 1A; ** *p* < 0.001).

In 3D culture, control A549 cells without AF293 conidia formed spheroids. However, A549 cells with AF293 conidia (1:10 and 1:100) formed incomplete spheroids, a trend that was more evident in the 1:100 group than in the 1:10 group (Figure 1B). The relative areas of A549 spheroids with AF293 conidia at ratios of 1:10 and 1:100 was 0.59- and 0.42-fold smaller than those of the control at day 7, respectively (Figure 1B; ** *p* < 0.001). The relative area of A549 spheroids with AF293 conidia at a ratio of 1:100 on day 7 was 0.716-fold smaller than that on day 3 (Figure 1B; ** *p* < 0.001). These results suggest that AF293 conidia suppress the growth of epithelial cells.

To detect the involvement of apoptosis in the growth suppression, the apoptotic reaction of caspase-3/7 was observed in spheroids with AF conidia. The signal in the spheroid with AF293 conidia challenge at a ratio of 1:10 was not significantly different; however, the signal at a ratio of 1:100 was 1.23-fold larger than that of control, suggesting that AF293 conidia induce luminal apoptosis (Figure 1C; * *p* < 0.01). These results suggest the involvement of apoptosis in the efficacy of growth suppression by AF293 conidia.

### 3.2. An RNA-Seq Analysis of Genes Induced by AF293 Conidia in A549 Cells

**Figure 2:**
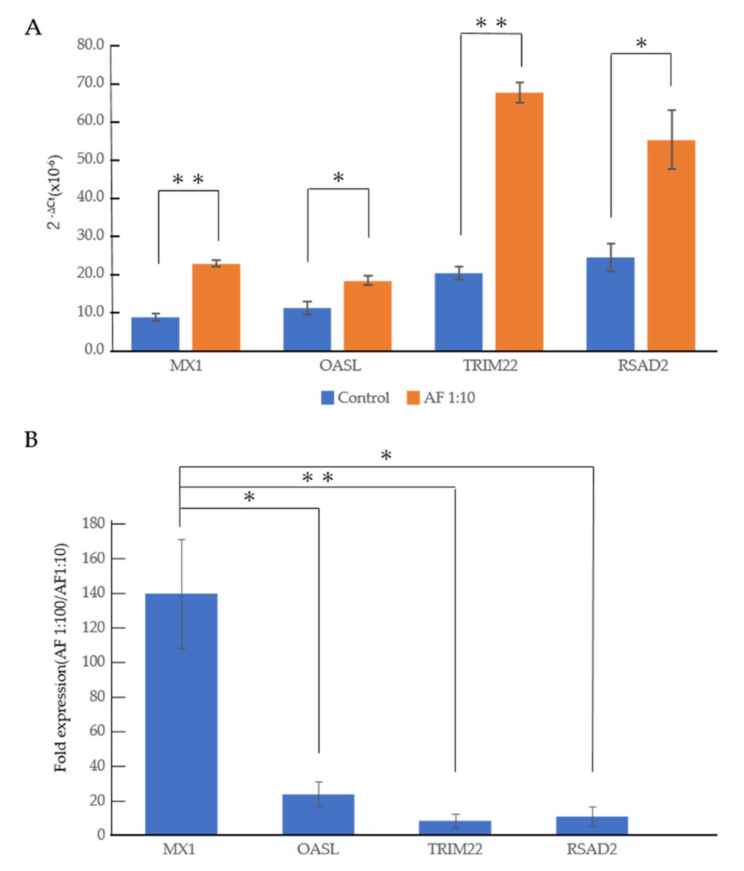
Heat-inactivated AF293 conidia increase the mRNA expression of MX1, OASL, TRIM22 and RSAD2 in A549 cells. (**A**) The mRNA expression of MX1, OASL, TRIM22 and RSAD2 in A549 cells with AF293 conidia at a ratio of 1:10 on day 3 in 2D culture. The X axis represents the expression value [2^−ΔCt^(×10^−6^)]. (**B**) The relative mRNA expression of MX1, OASL, TRIM22 and RSAD2 in A549 cells with AF293 conidia at ratios of 1:100 in comparison to those at a ratio of 1:10 on day 3 in 2D culture. The mean ± SD from three biological replicates is shown. * *p* < 0.01, ** *p* < 0.001.

**Figure 3:**
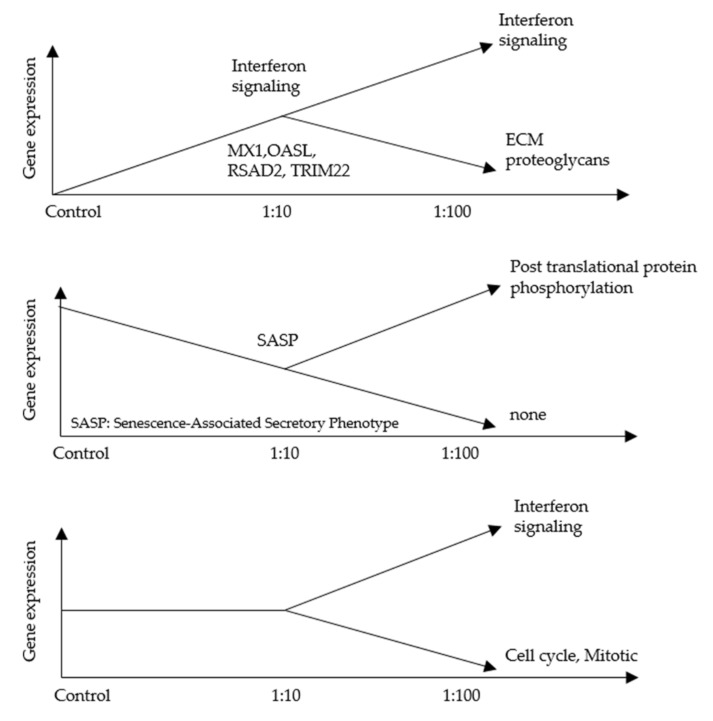
A schematic representation of the relative mRNA expression, ontology and related genes from RNA-seq data of A549 cells with AF293 conidia at ratios of 1:10 and 1:100 on day 3 in 2D culture. The X axis represents the gene expression levels.

To explore the effect of AF293 conidia on A549 cells, RNA-seq was performed. We selected genes showing a 2-fold increase of mRNA expression at a ratio of 1:10 compared with the control by AF293 conidia challenge (Supplementary Table S1). We found that 10 genes showed a 2-fold increase in mRNA expression at a ratio of 1:100 compared with that of 1:10 by the AF293 conidia challenge (Table 1). An ontology analysis of these genes revealed that the expression of genes associated with interferon (IFN) signaling, including MX1, OASL, RSAD2 and TRIM22, were increased by AF293 conidia (Table 2). Real-time quantitative reverse transcription-polymerase chain reaction (qRT-PCR) was performed to confirm the mRNA expression of these genes (Figure 2). As shown in the RNA-seq analyses (Table 1), the mRNA expression of these genes was significantly increased at a ratio of 1:10 in comparison to control by an AF293 conidia challenge (Figure 2A; * *p* < 0.01; ** *p* < 0.001), and was further increased at a ratio of 1:100 in comparison to the ratio of 1:10 by the AF293 conidia challenge (Figure 2B). The increased expression of MX1 is dominant in comparison to that of OASL (* *p* < 0.01), RSAD2 (* *p* < 0.01), and TRIM22 (** *p* < 0.001), suggesting the critical roles of MX1 during AF293 conidia challenge. Notably, ontology analyses revealed that the downregulated genes at a ratio of 1:100 in comparison to the ratio of 1:10 by the AF293 conidia challenge was associated with the cell cycle among genes for which the expression at a ratio of 1:10 was not changed from that of the control (Figure 3, Supplementary Table S2), suggesting an association with the induction of apoptosis by AF conidia in A549 cells.

### 3.3. AF293 Conidia Did Not Increase the Expression of Epithelial-Mesenchymal Transition (EMT)-Related Genes in A549 Cells

**Figure 4:**
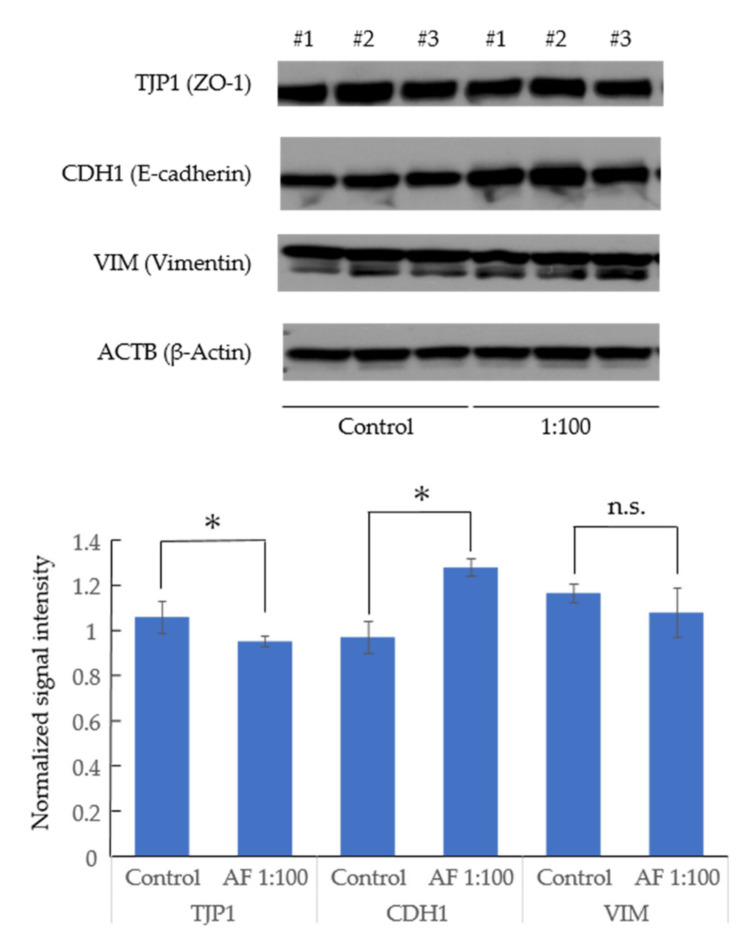
The assessment of the expression of epithelial-mesenchymal transition (EMT)-related markers in A549 cells by Western blotting. The expression of the indicated proteins in the lysates was examined using specific antibodies for TJP1 (ZO-1), CDH1 (e-cadherin), VIM (vimentin) and ACTB (β-actin). Actin was used as a loading control. The left-half columns (#1–3) show the findings for the A549 cells in the control group. The right-half columns (#1–3) show the findings for the A549 cells with AF293 conidia at a ratio of 1:100. AF293 conidia did not increase the expression of EMT-related genes in A549 cells. Data are representative of three independent experiments. The lower panel represents the signal intensity normalized by the intensity of ACTB. The mean ± SD from three biological replicates is shown. * *p* < 0.05. n.s., not significant.

To investigate whether or not heat-inactivated AF conidia causes EMT in A549, we cultured A549 with AF293 conidia at a ratio of 1:100 in 2D culture. The expression of ZO-1 (an epithelial marker) showed a 0.89-fold decrease, while that of e-cadherin (another epithelial marker) was increased 1.32-fold, suggesting that the change in epithelial markers is inconsistent. There was no significant change in the expression of EMT-associated proteins, vimentin (Figure 4), suggesting that AF293 conidia did not affect the expression of EMT-related genes in A549 cells.

### 3.4. AF293 Conidia Promoted the Growth of Mouse NIH/3T3 Cells

**Figure 5:**
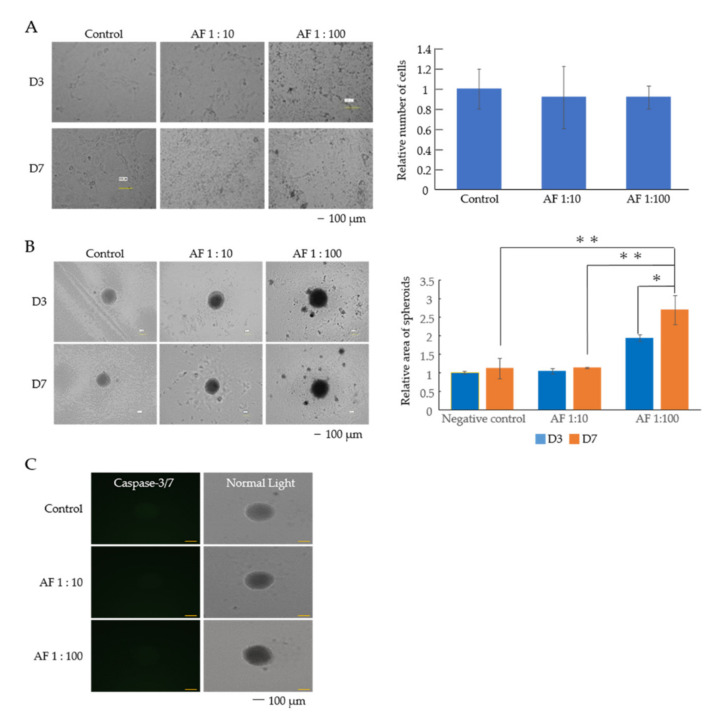
Heat-inactivated AF293 conidia promoted the growth of mouse NIH/3T3 cells. (**A**) Morphological changes in mouse NIH/3T3 cells with AF293 conidia at ratios of 1:10 and 1:100 on days 3 and 7 in 2D culture. AF 1:10 and AF 1:100 represents the number of AF293 conidia are 10- and 100-fold higher than that of mouse NIH/3T3 cells. D3: day 3, D7: day 7, scale bar: 100 μm. Right panel represents the relative number of cells. The mean ± SD from three technical replicates is shown. (**B**) Morphological changes and the relative area of mouse NIH/3T3 cells spheroids with AF293 conidia at ratios of 1:10 and 1:100 on day 7 in 3D culture. Right panel represents the area of spheroids. The mean ± SD from three technical replicates is shown. * *p* < 0.001, ** *p* < 0.01. (**C**) Representative signals for caspase-3/7 in mouse NIH/3T3 spheroids from three technical replicates (Scale bar: 100 μm). All data are representative of three independent experiments.

To compare the effects of AF293 on A549 cells, we examined the effects of AF293 on mouse NIH/3T3 cells. 3T3 cells were cultured with heat-inactivated AF293 conidia in the same way. In 2D culture, the growth of 3T3 cells with or without heat-inactivated AF293 (1:10 and 1:100) did not differ to a statistically significant extent (Figure 5A).

In 3D culture, control 3T3 cells formed spheroids; 3T3 cells with AF293 (1:100) formed even larger spheroids than those in the control group. (Figure 5B). The relative areas of 3T3 spheroids with AF293 conidia at 1:100 was 2.41- and 2.38-fold larger than those of the control and those at 1:10 on day 7, respectively (Figure 5B; ** *p* < 0.01). The relative area of 3T3 spheroids with AF293 conidia at a ratio of 1:100 on day 7 was 1.39-fold larger than that on day 3 (Figure 5B; * *p* < 0.1). These results suggest that AF293 conidia promoted the growth of 3T3 cells in a 3D-specific way. The signal of caspase-3/7 was not completely detected in any spheroids with AF293 conidia (Figure 5C).

### 3.5. An RNA-Seq Analysis of Genes Induced by AF293 Conidia in NIH/3T3 Cells

**Figure 6:**
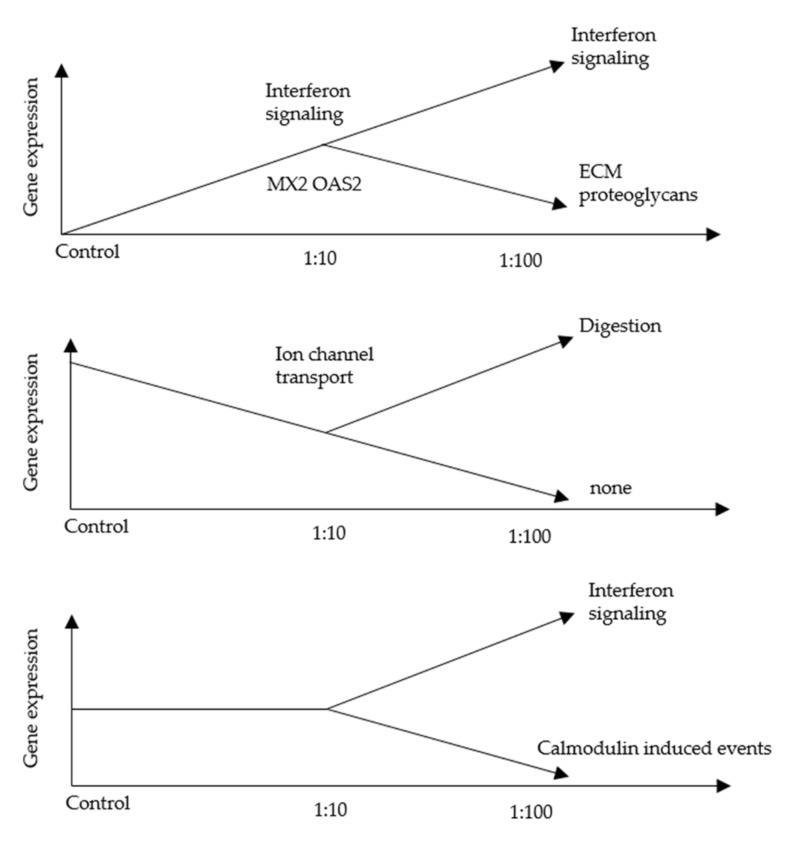
A schematic representation of the relative mRNA expression, ontology and related genes from RNA-seq data of NIH/3T3 cells with AF293 conidia at ratios of 1:10 and 1:100 on day 3 in 2D culture. The X axis represents the of gene expression levels.

To explore the effect of AF293 conidia on NIH/3T3 cells, RNA-seq was performed. We selected genes for which the mRNA expression showed a 2-fold increase at a ratio of 1:10 in comparison to control in response to an AF293 conidia challenge (Supplementary Table S3). We found that 25 genes showed for which the mRNA expression showed a 2-fold increase at a ratio of 1:100 in comparison to the expression at 1:10 in response to an AF293 conidia challenge (Table 3). An ontology analysis of these genes revealed that the expression of genes associated with IFN signaling, including Oas2, Mx2 and Rsad2, was increased by AF293 conidia (Table 4). Notably, the ontology analyses revealed that, among the genes for which the expression did not change between a ratio of 1:10 and control, the genes that were downregulated at a ratio of 1:100 in comparison to a ratio of 1:10 in response to an AF293 conidia challenge were not associated with the cell cycle (Figure 6), suggesting that AF conidia does not induce apoptosis in NIH/3T3 cells.

## 4. Discussion

In the present study, we performed 2D and 3D culture with heat-inactivated AF293 conidia using human lung epithelial A549 cells and found that AF293 conidia reduced the proliferation of A549 cells in both 2D and 3D cultures. Furthermore, we performed RNA-seq using 2D-cultured A549 cells with AF293 conidia and detected early response genes associated with IFN signaling, such as MX1, OASL, RSAD2 and TRIM22. Notably, type-I IFN acts on cells during viral infections to inhibit viral replication [22]. Indeed, a recent study revealed the expression of these genes in A549 cells infected by influenza virus [23].

An ontology analysis failed to show any apparent association between AF293 conidia and apoptosis; however, cell cycle genes were downregulated by high concentrations of AF293 conidia; Figure 3, Supplementary Table S2). This suggests that apoptosis was induced by a checkpoint mechanism. Furthermore, EMT was not clearly observed (Figure 4), suggesting that a longer exposure or higher concentration administration may be needed for EMT induction.

It was recently hypothesized that idiopathic pulmonary fibrosis might be a neoproliferative disorder of the lung because this disease exhibits several pathogenic features similar to cancer [24]. Surprisingly, our study showed that AF conidia increase the growth of NIH/3T3 spheroids despite no significant change in 2D culture (Figure 5). Ontology analyses of RNA-seq data shows similar patterns with those seen in A549 cells. For example, genes associated with IFN signaling, such as Oas2, Mx2 and Rsad2, are upregulated by dose dependent manner. However, downregulation of genes associated with cell cycle is not observed. Humans possess two MX genes, MX1 and MX2, with a high level of homology [25]. MX protein is considered as a key mediator of the IFN-induced antiviral response. MX proteins contain the typical GTP-binding motif and show significant homology to the dynamin family of GTPases. Strong interaction of MX proteins with tubulin suggests that Mx proteins could be involved in mitosis [26,27]. However, roles of MX proteins in proliferation and apoptosis are inconsistent. Indeed, the increased expression of MX1 is associated with liver fibrosis and high MX1 protein expression is associated with increased proliferation and poor prognosis in breast cancer [28,29]. On the other hand, the gain of MX1 expression in in human prostate cancer LNCaP cells resulted in cell cycle arrest [26]. Regarding MX2 protein, overexpression of MX2 in primary melanoma reduces in vivo proliferation partially through inhibition of AKT activation. However, in a subset of melanoma cell lines with high endogenous MX2 expression where downregulation of MX2 leads to reduced proliferation [30]. Our results also demonstrate that AF conidia promote 3T3 proliferation in 3DF but not in 2D. The structure and distribution of the cytoskeleton is profoundly influenced by the dimensionality of a cell’s environment [31]. These results suggest that the binding between MX proteins and cytoskeleton is influenced by 2D or 3D culture and cellular response by AF conidia is context-dependent possibly through critical roles of MX proteins for the cytoskeleton. Interestingly, the increased expression of MX1 autoantibody in serum is associated with a good prognosis in patients with idiopathic interstitial pneumonia [32], suggesting that the inhibition of MX proteins is a possible strategy for preventing fibrotic lung diseases.

In conclusion, our study showed that high concentrations of AF293 conidia induce A549 apoptosis along with IFN inducible genes, such as MX1. In addition, AF293 conidia increase NIH/3T3 proliferation possibly through IFN signaling, such as MX2 induction. These results suggest the role of MX proteins during colonization. Although several mechanisms for epithelial apoptosis and fibroblastic proliferation were postulated [33], whether or not different responses of these two types of cell to AF293 conidia are involved in the development of pulmonary fibrosis remains unclear. More detailed analyses are expected to help clarify the spatiotemporal regulation of AF293 conidia, resulting in further understanding of the pathogenesis of *Aspergillus* spp. in pulmonary fibrosis.

## Figures and Tables

**Table 1:** Genes upregulated by AF293 conidia.

Gene Symbol	1:10/Control	1:100/Control	1:100/1:10
MX1	2.53020134	189.2080537	74.77984085
RSAD2	2.48148148	75.2962963	30.34328358
OASL	2.88034188	50.98290598	17.70029674
CMPK2	2.19444444	33.41666667	15.2278481
PARP10	2.03125	19.90625	9.8
TRIM22	2.81481481	16.65432099	5.916666667
RARRES3	3.39285714	14.46428571	4.263157895
SAMD9L	2.60608622	10.30092984	3.952643529
SSPO	2.075	7.4625	3.596385542
KNDC1	2.56603774	6.132075472	2.389705882
GOLGA8EP	2.11111111	4.555555556	2.157894737
AC068533.7	3.34883721	6.953488372	2.076388889

**Table 2:** Ontology analysis results.

Name of Pathway	Set Size	Candidates Contained	*p*-Value	Genes
Interferon signaling	158	4 (2.5%)	<0.0001	MX1, OASL, TRIM22, RSAD2
Interferon alpha/beta signaling	70	3 (4.3%)	<0.0001	MX1, OASL, RSAD2
Cytokine signaling in immune system	458	4 (0.9%)	<0.0001	MX1, OASL, TRIM22, RSAD2
Interferon gamma signaling	94	2 (2.1%)	0.001	OASL, TRIM22
Immune system	1840	4 (0.2%)	0.001	MX1, OASL, TRIM22, RSAD2

**Table 3:** Upregulated genes by Af293 conidia.

Gene Symbol	1:10/Control	*p*-Value	1:100/Control	*p*-Value	1:100/1:10	*p*-Value
Oas2	17.6	0.01418642	227.0666667	0.000142248	12.90151515	0.000217291
Oasl2	12.8181818	0.00105008	153.6439394	2.98985 × 10^−8^	11.98640662	1.97743 × 10^−7^
Oas1a	3.90163934	0.00115744	44.29508197	4.56841 × 10^−5^	11.35294118	6.24434 × 10^−5^
Mx2	9.27692308	0.00694089	103.4923077	1.36501 × 10^−6^	11.15588723	4.53558 × 10^−6^
Oas1g	3.66666667	0.00878991	35.48888889	8.18609 × 10^−5^	9.678787879	0.000126154
Usp18	2.55406912	0.00924884	20.79487179	9.33815 × 10^−5^	8.14185945	0.000131795
Zbp1	2.7	0.08168567	21.74	0.000237643	8.051851852	0.000479164
Isg15	4.02118003	0.01702177	30.3494705	9.91865 × 10^−5^	7.547404063	0.000208583
Bst2	3.45379024	0.00064043	25.64382139	3.26371 × 10^−6^	7.424834636	6.17672 × 10^−6^
Rtp4	3.24145786	0.01492393	21.29840547	9.5736 × 10^−5^	6.570625439	0.000206501
Gbp3	4.82394366	0.004038	29.6971831	0.000166256	6.15620438	0.000345417
Irgm2	7.52132701	0.005725	45.36966825	4.0298 × 10^−5^	6.032136106	0.000125601
Rsad2	2.56321839	0.00226457	14.55172414	5.23578 × 10^−5^	5.677130045	8.98531 × 10^−5^
Apol9a	2.4	0.00182007	12.2691358	0.000515447	5.112139918	0.000880427
Ifit3	3.18018018	0.01196764	16.25675676	3.98466 × 10^−6^	5.111898017	3.48212 × 10^−5^
Xaf1	6.21891419	0.00600125	30.08231173	5.8742 × 10^−6^	4.83722895	6.10851 × 10^−5^
Irf7	2.27311828	0.00105694	10.51397849	0.000500941	4.625354778	0.000872438
Oas1b	4.12820513	0.02932599	18.07692308	7.73289 × 10^−5^	4.378881988	0.000548655
Ifit1	4.65353038	0.00588437	18.42857143	2.18323 × 10^−7^	3.960127029	4.76394 × 10^−5^
Lgals3bp	2.61022273	0.00030663	9.311536265	0.000139853	3.56733399	0.000351664
Helz2	2.16917969	7.7487 × 10^−5^	6.965140407	8.40955 × 10^−7^	3.210955934	2.64734 × 10^−6^
Ddx58	2.93550577	0.00063444	9.393075356	1.45365 × 10^−5^	3.199814986	7.08851 × 10^−5^
Parp9	2.15489614	0.00044662	5.903264095	7.46111 × 10^−6^	2.739465712	2.5749 × 10^−5^
Irf9	2.77596342	0.00099975	7.331809275	2.31458 × 10^−5^	2.641176471	0.000182173
Apol8	2.20883534	0.00066116	4.977911647	0.000122632	2.253636364	0.000723058

**Table 4:** Ontology analysis of genes shown in Table 3.

Name of Pathway	Set Size	Candidates Contained	*p*-Value
Interferon Signaling	158	13 (8.2%)	1.76 × 10^−22^
Interferon alpha/beta signaling	70	11 (15.7%)	5.88 × 10^−22^
Cytokine Signaling in Immune system	458	13 (2.8%)	2.30 × 10^−16^
Immune System	1840	14 (0.8%)	5.06 × 10^−10^
ISG15 antiviral mechanism	30	4 (13.3%)	5.38 × 10^−8^
Antiviral mechanism by IFN-stimulated genes	30	4 (13.3%)	5.38 × 10^−8^
Interferon gamma signaling	94	4 (4.3%)	5.69 × 10^−6^
DDX58/IFIH1-mediated induction of interferon-alpha/beta	56	3 (5.4%)	4.94 × 10^−5^
TRAF3-dependent IRF activation pathway	13	2 (15.4%)	0.000125
TRAF6 mediated IRF7 activation	17	2 (11.8%)	0.000217
Negative regulators of DDX58/IFIH1 signaling	23	2 (8.7%)	0.000401
Cytosolic sensors of pathogen-associated DNA	51	2 (3.9%)	0.00198
Innate Immune System	1077	5 (0.5%)	0.00985

## Data Availability

Not applicable.

## Supplementary Materials

The following are available online at https://www.mdpi.com/article/10.3390/microorganisms9071435/s1.
